# Novel *Escherichia coli* RF1 mutants with decreased translation termination activity and increased sensitivity to the cytotoxic effect of the bacterial toxins Kid and RelE

**DOI:** 10.1111/j.1365-2958.2008.06510.x

**Published:** 2008-10-31

**Authors:** Elizabeth Diago-Navarro, Liliana Mora, Richard H Buckingham, Ramón Díaz-Orejas, Marc Lemonnier

**Affiliations:** 1 Department of Molecular Microbiology, Centro de Investigaciones Biológicas CSIC, Ramiro de Maeztu 9E-28040 Madrid, Spain; 2 IBPC, CNRS UPR 9073, 13 rue Pierre et Marie CurieF-75005 Paris, France

## Abstract

Novel mutations in *prfA*, the gene for the polypeptide release factor RF1 of *Escherichia coli*, were isolated using a positive genetic screen based on the *parD* (*kis*, *kid*) toxin–antitoxin system. This original approach allowed the direct selection of mutants with altered translational termination efficiency at UAG codons. The isolated *prfA* mutants displayed a ∼10-fold decrease in UAG termination efficiency with no significant changes in RF1 stability *in vivo*. All three mutations, G121S, G301S and R303H, were situated close to the nonsense codon recognition site in RF1:ribosome complexes. The *prfA* mutants displayed increased sensitivity to the RelE toxin encoded by the *relBE* system of *E. coli*, thus providing *in vivo* support for the functional interaction between RF1 and RelE. The *prfA* mutants also showed increased sensitivity to the Kid toxin. Since this toxin can cleave RNA in a ribosome-independent manner, this result was not anticipated and provided first evidence for the involvement of RF1 in the pathway of Kid toxicity. The sensitivity of the *prfA* mutants to RelE and Kid was restored to normal levels upon overproduction of the wild-type RF1 protein. We discuss these results and their utility for the design of novel antibacterial strategies in the light of the recently reported structure of ribosome-bound RF1.

## Introduction

Translation termination is an important stage in protein synthesis which leads to the release of the newly synthesized polypeptide chain from the ribosome. This is initiated when a termination codon in mRNA enters the ribosomal A site and is recognized by a class 1 release factor (RF). In bacteria, translation termination requires the active contribution of two protein release factors, RF1 and RF2 (Scolnick *et al*., 1968); these factors recognize, respectively, and with different affinity UAA/UAG or UAA/UGA termination codons at the decoding centre of the ribosomal A site. A conserved tripeptide motif GGQ in RF1 or RF2 interacts with the peptidyl transfer centre (PTC) (Frolova *et al*., 1999) and activates the hydrolysis of the ester bond between the polypeptide chain and the tRNA at the PTC, thereby releasing the newly synthesized polypeptide chain.

Specific recognition of termination codons by RF1 and RF2 involves specific tripeptide motifs, PxT for RF1 or SPF for RF2 (Ito *et al*., 2000), which are present in a central domain of the proteins. The tripeptide motifs were first suggested to be responsible for recognizing the last two nucleotides of the three stop codons (Ito *et al*., 2000), but recent structural studies indicate that they interact with the first two nucleotides (Laurberg *et al*., 2008) and explain in part the remarkable accuracy with which the first stop codon nucleotide, an invariant uridine, is recognized (Freistroffer *et al*., 2000). Discrimination against purines in the first stop codon position implicates residues near the tip of a helical finger (α-helix 5) present in the central domain (Laurberg *et al*., 2008). Two residues near the PxT motif, Q185 and T198 in *Escherichia coli* RF1, discriminate against pyrimidines in the third stop codon position (Laurberg *et al*., 2008). Interactions of RF1 or RF2 with a third factor, RF3-GTP or GDP, at the GTP-associated centre of the 50S ribosomal subunit, are also required for the final detachment of the class 1 RFs from the ribosome (Freistroffer *et al*., 1997; Zavialov *et al*., 2001; 2002; Gao *et al*., 2007).

A more detailed understanding of the role of the GGQ motif conserved in class I RFs has been provided by crystal structures of RF1 and RF2 in complex with the ribosome (Petry *et al*., 2005; Laurberg *et al*., 2008), and by molecular dynamics simulations of peptide release (Trobro and Aqvist, 2007). The GGQ motif, together with A76 of the P-site tRNA, is instrumental in co-ordinating the water molecule that initiates a nucleophilic attack on the peptidyl-tRNA ester bond. Following the detachment of the RF from the ribosome the subsequent action of the ribosomal releasing factor RRF and the elongation factor EF-G (Karimi *et al*., 1999; Gao *et al*., 2007) leads to the de-assembling of the ribosomal subunits, which can then be recycled and reassembled in a new translation initiation event.

Pausing of a bacterial ribosome due to limitations in tRNA can lead to cleavage of mRNA at a site within or immediately adjacent to the A-site codon; the ribosome participates in this cleavage in an, as yet, undefined way (Hayes and Sauer, 2003; Sunohara *et al*., 2004). Breaks at UAG and UGA stop codons induced by ribosome stalling due to the reduced activity of RF1 have been reported (Li *et al*., 2007). Breaks in the mRNA at UAG termination codons at the A site can also be induced by the RelE toxin encoded by the bacterial toxin–antitoxin (TA) system *relBE*. The cleavage occurs preferentially between the second and third nucleotides of UAG termination codons in the ribosomal A site implying the concerted action of the RelE toxin and the ribosome (Pedersen *et al*., 2003). The RelE toxin is activated under amino acid or carbon source limitation by a mechanism which involves the enhanced decay of the RelB antitoxin protein (Gerdes *et al*., 2005). Consistent with this, RF1, which preferentially recognizes this termination codon at the A site, can protect against the RelE cleavage *in vitro* (Pedersen *et al*., 2003). Cleavage of mRNA in all the above cases leads to the stalling of the ribosome at the end of the non-stop mRNA, and can eventually inhibit protein synthesis as in the case with RelE. Stalled ribosomes are released by the intervention of transfer-messenger RNA (tmRNA), which provides a new stop codon and tags potentially harmful truncated peptides for rapid degradation (Moore and Sauer, 2007); this, in turn, feeds amino acid pools [see Discussions by Gerdes *et al*. (2005) and by Condon (2006)].

The *kis–kid* antitoxin–toxin system of plasmid R1 codes for an unstable antitoxin Kis and a stable toxin Kid. Kid, like RelE, is also a specific endoribonuclease and an inhibitor of translation, but unlike RelE, Kid can cleave RNA in the absence of ribosomes and does so at the core sequence UA(A/C) preferentially but not exclusively in single-stranded regions (Zhang *et al*., 2004; Munoz-Gomez *et al*., 2005). Cleavage occurs by a mechanism similar to the RNase A and RNase T1, involving the Uracyl 2′-OH group and producing two fragments, with a 2′:3′-cyclic phosphate group at the uracyl and a free 5′-OH group at the adenosine respectively. Cleavage of RNA in the absence of ribosomes can also introduce non-stop mRNA in the translation machinery, a proposal that is consistent with the increased sensitivity to the Kid toxin observed in tmRNA-deficient strains (Munoz-Gomez, 2004).

While searching for *E. coli* mutations affecting the toxic activity of the Kid protein, we developed a genetic screen that was based on the selection of bacteria that could survive in the presence of a mutant *parD* locus encoding a functional Kid toxin and a truncated and inactive antitoxin resulting from an amber termination mutation in the *kis* antitoxin gene (Bravo *et al*., 1988). As a consequence, the isolation of suppressors leading to the efficient bypass of the amber mutation in *kis* was one anticipated response. Unexpectedly, however, these mutations were found to be novel and to be exclusively targeted to the *prfA* gene encoding the RF1 protein. Our work reported here shows that these mutations reveal novel functional connections between the activity of bacterial toxins and translation termination factors.

## Results

### Isolation of novel *prfA* mutants in *E. coli*

Our work was initially focused on a search for *E. coli* mutants resistant to the cytotoxic activity of Kid. For this purpose, we used a double selection approach inspired by the work by Bernard and Couturier (1992), which led to the isolation of mutations in the *gyrA* gene encoding the A subunit of DNA gyrase, the bacterial target of the CcdB toxin. Two compatible plasmids were employed, pKK1120 and pAB1120, that confer resistance to tetracycline and kanamycin, respectively, and which both contain the mutant *parD* operon *kis74* (amber), *kid*+ (Table 1). In the absence of suppression of the *kis74* mutation, an inactive form of the Kis antitoxin is produced, thus leading to a Kid-dependent inhibition of colony formation. In a first round of selection, *E. coli* cells treated with the mutagen 2-aminopurine were transformed with the pKK1120 plasmid and cultured in medium containing tetracycline. To enrich for potential Kid-resistant mutants, a second round of selection was carried out by transforming the cells with the pAB1120 plasmid and plating on medium supplemented with kanamycin. To discard possible mutations affecting either the *kid* gene itself or its regulatory elements present in the plasmid, the toxicity of the resident plasmids was assessed by extracting the plasmid DNA and retransforming fresh wild-type *E. coli* cells. Mutants that exhibited growth despite containing a toxic plasmid were kept for further characterization.

**Table 1:** Plasmids used and constructed in this work.

Plasmid	Relevant features	Reference
pAB1120	R1, *copB*^-^, *parD*[*kis74*, *kid*(*wt*)], *kan*	Bravo *et al.*
pKK1120	pACYC184-derivative plasmid, parD [*kis74*, *kid*(*wt*)], *tet*	This work
pBAD18	pBR322-derivative expression vector, *amp*	Guzman *et al*. (1995)
pELI01	pBAD18, P*ara::prfA121*, *amp*	This work
pELI02	pBAD18, P*ara::prfAwt*, *amp*	This work
pELI03	pBAD18, P*ara::prfA301*, *amp*	This work
pELI04	pBAD18, P*ara::prfa304*, *amp*	This work
pELI05	pFUS2, P*ara::kis*, *kan*	This work
pELI06	pFUS2, P*ara::kis74*, *kan*	This work
pELI07	pNDM220, P*lac::relE*, *amp*	This work
pELI08	pFUS2, P*ara::prfAwt*, *kan*	This work
pELI09	pFUS2, P*ara::relB*, *kan*	This work
pET80	parD [*kis*(*wt*), *kid*(*wt*)], *kan*	Bravo *et al*. (1988)
pMLM1	mini-F, *repFIA*+, *sop*+, *cat*	Lemonnier *et al*. (2000)
pFUS2	pFUS-derivative expression vector, *kan*	Lemonnier *et al*. (2000)
pNDM220	miniR1-derivative expression vector, *amp*	Gotfredsen and Gerdes (1998)
pSS100	pNDM220, P*lac*::*kid*, *amp*	Santos_Sierra *et al*. (2002)

The mutations were mapped to the region between 22 and 32 min on the *E. coli* chromosome using a collection of different Hfr-Tn*10* strains (data not shown). To refine the genetic mapping of the mutations, the mutant strains were used as recipients in P1 transduction experiments performed using different donor strains bearing Tn*10* transposon insertions at regular intervals between minutes 22 and 32 on the chromosome. For one of the mutants we observed that the pAB1120-resistance phenotype was lost at high frequency upon transduction from a donor containing the Tn*10* at position 26.61 min. In order to isolate the mutant gene, fragments of the chromosome of the mutant strain (hereafter the KR19 strain), generated by partial Sau3A restriction digestion, were cloned into a low-copy-number mini-F, pMLM1 (Lemonnier *et al*., 2000). *E. coli* cells were transformed with this library, pooled and subsequently transformed with the pAB1120 plasmid. Mini-F plasmids carrying fragments that conferred resistance to pAB1120 were isolated and their chromosomal insert sequenced. All the sequenced fragments consisted in a chromosomal region enclosing the *prfA* gene encoding polypeptide release factor RF1. The localization of this gene at 27.2 min in the *E. coli* chromosome was consistent with the results of the Hfr mapping. Subsequently, the sequencing of the *prfA* region of KR19 and of several independent mutants isolated by this approach revealed that in all cases mutations in the *prfA* gene could be detected. Four independent mutants, hereafter called KR2, KR4, KR17 and KR19, were kept for subsequent studies. Sequencing analysis revealed that the mutations consisted of single-base G-C or A-T transitions, as expected from a 2-aminopurine mutagenic treatment (Ronen, 1980). Strains KR2 and KR19 were found to bear the same mutation, *prfA301*, which led to a Gly to Ser amino acid change at position 301 of RF1. Strain KR4 carried the mutation *prfA303*, in which an Arg has changed to Ser at position 303 of RF1. Finally, KR17 strain carried the mutation *prfA121*, a Gly to Ser change at position 121 of RF1.

### The *prfA* mutants are unable to complement a *prfA(ts)* mutation unless they are overexpressed

**Fig. 1:**
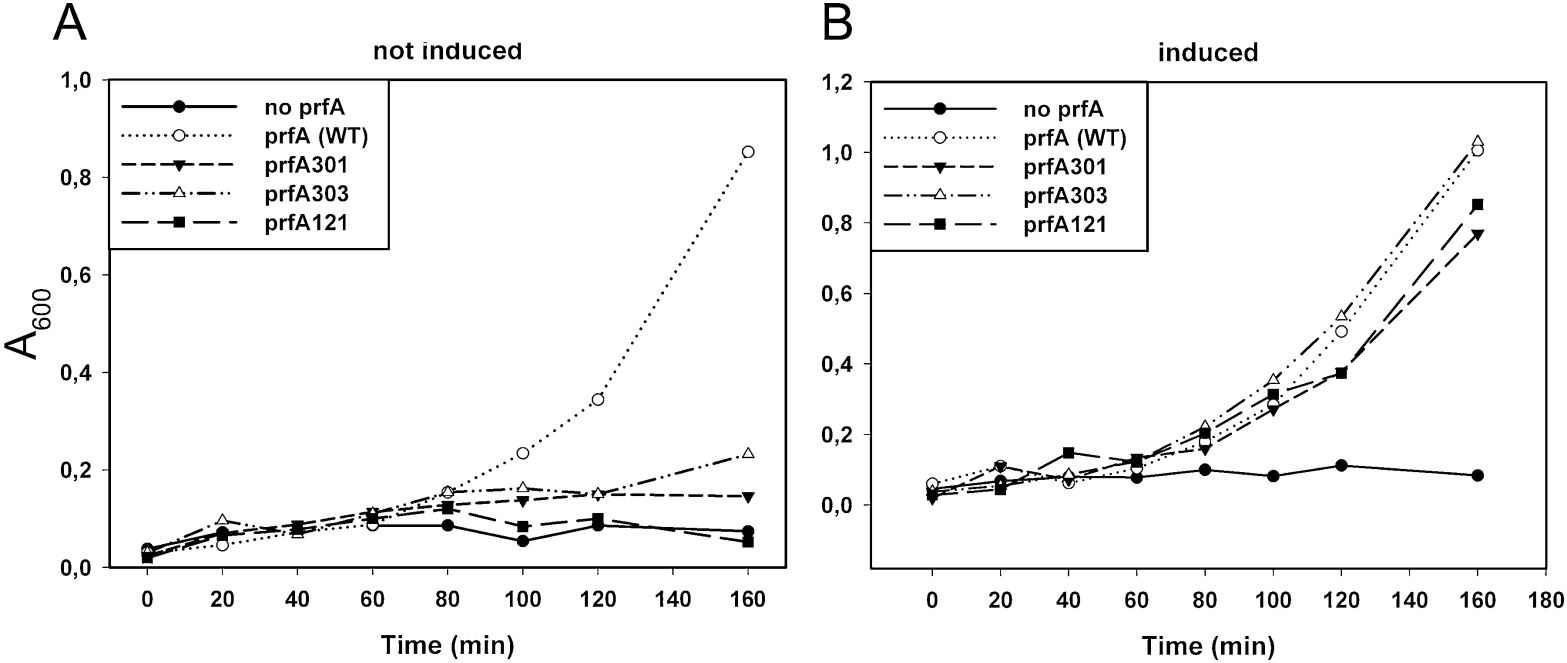
Complementation of a temperature-sensitive *prfA* mutation. MRA8 [*prfA1*(*ts)*] strain was transformed with the plasmids pELI01 (*prfA*121), pELI02 (*prfA*wt), pELI03 (*prfA*301) and pELI04 (*prfA*303). Expression of the *prfA* alleles in these plasmids was placed under the control of a promoter whose activity could be induced by adding arabinose into the medium. A. Uninduced cultures were grown at 42°C for up to 160 min. B. Same experiment but arabinose (1% final) was present in the medium to induce the expression of the different *prfA* alleles.

To assess whether the isolated *prfA* mutants were affected in their translation termination activity *in vivo*, we performed a complementation assay using a conditional temperature-sensitive *E. coli* mutant, MRA8 *prfA1(ts)* (Ryden and Isaksson, 1984; Zhang *et al*., 1994). The strain was transformed with the pBAD18 plasmid derivatives pELI01, pELI02, pELI03 and pELI04, which carried the alleles *prfA121*, *prfA wt*, *prfA301* and *prfA303*, respectively, under the control of the arabinose-inducible promoter pBAD (Guzman *et al*., 1995). MRA8 was also transformed with pBAD18 as a control. At basal expression levels (i.e. in the absence of arabinose), wild-type *prfA* expressed from pELI02 was able to complement the *prfA1(ts)* mutation at the non-permissive temperature of 42°C, whereas none of the mutants was able to do so (Fig. 1, left). When expression of the *prfA* alleles was induced by adding arabinose to the medium, the growth of strain MRA8 at 42°C was significantly recovered for all the mutants tested (Fig. 1, right). Since overexpression of the mutant proteins was able to achieve complementation, these results indicated that the mutations significantly decreased, but did not abolish, the efficiency of translational termination by RF1.

### Decreased translation termination *in vivo* leads to readthrough of stop codons

**Fig. 2:**
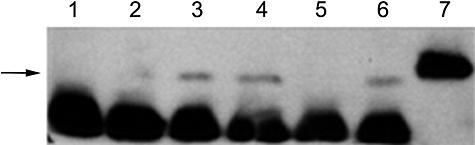
*In vivo* readthrough of the *kis74* amber mutation in KR19 strain. Western blot analysis was performed using different cell extracts as described in *Experimental procedures*. The black arrow indicates full-length Kis protein. Lanes 1, 3 and 5, cell extracts from strain MC1061 bearing pFUS2, pELI05 (*kis*) and pELI06 (*kis74*) plasmids, respectively; lanes 2, 4 and 6, cell extracts from strain KR19 carrying pFUS2, pELI05 and pELI06 plasmids, respectively; lane 7, purified his-tagged Kis protein. The thick band at the bottom of the gel is a non-specific product present in whole-cell lysates.

Since the mutations affected RF1, which specifically recognizes UAG (amber) stop codons, a likely explanation for the phenotype of resistance displayed by the mutants was the readthrough of the *kis74* amber mutation, which would allow the production of an active, full-length antitoxin. Wild-type *E. coli* cells and KR19 mutant bacteria were transformed with either the plasmid vector pFUS2, or its derivatives pELI05 and pELI06 bearing the wild-type *kis* and the mutant *kis74* alleles, respectively, placed under the control of the arabinose-dependent pBAD promoter (Guzman *et al*., 1995). Following overexpression of the *kis* alleles by addition of arabinose to the cultures, cell lysates were prepared and analysed by Western blot using anti-Kis antibodies (Fig. 2). A band corresponding to the full-length Kis protein was detected in the lysates obtained from wild-type and KR19 cells carrying the pELI05 (*kis*+) plasmid. This band was not present in lysates of wild-type cells containing pELI06, which produced a Kis protein of a lower size resulting from the truncation of the protein at site 74. Note that this Kis74 protein could not be well detected because it co-migrated with a lower non-specific band. In strain KR19 containing pELI06, readthrough of the stop codon at position 74 yielded a Kis protein of higher molecular weight, most likely a full-length protein, indicating a decreased *in vivo* efficiency of translation termination by the mutant RF1 protein at UAG stop codons. Similar results were also obtained for the KR17 mutant (data not shown).

### RF1 mutations strongly reduce the specific termination activity of RF1 at UAG codons

**Fig. 3:**
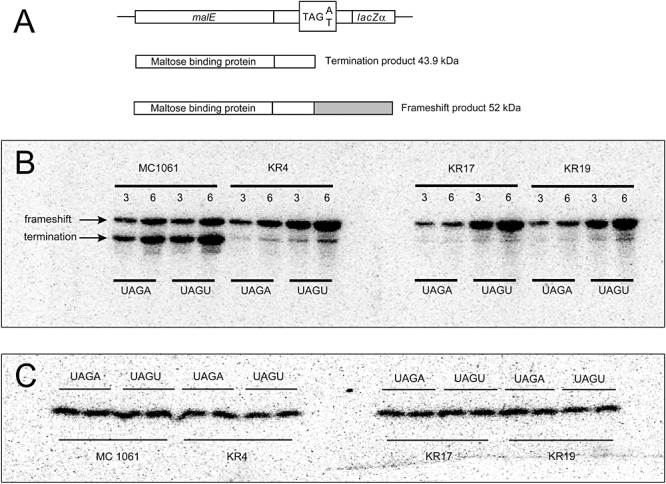
Efficiency and levels of RF1 in *prfA* mutant strains *in vivo*. Termination was measured by competition with frameshifting at the shifty site present in the *E. coli prfB* gene (Poole *et al*., 1995). A. A region of 23 nucleotides from the *prfB* gene around the frameshift site was fused to the *malE* gene present in a plasmid under the control of the P-tac promoter. Frameshifting (+1) of the ribosome paused at the in-frame stop signal allows translation to continue into a sequence derived from the *lacZ* gene. The wild-type tetranucleotide stop signal TGAC was replaced by the tetranucleotides TAGA or TAGU. B. Expression of the MalE fusion products was induced *in vivo* in the parental strain MC1061 and each of the mutant strains KR4, KR17 and KR19 with IPTG for 2 h. The protein products of termination and frameshifting present in 3 or 6 μl of cell extract (see *Experimental procedures*) were separated on SDS gels and visualized by Western blotting using anti-MalE antibodies and ^125^I-labelled protein A. C. The amounts of RF1 in parental and *prfA* mutant strains were determined by quantitative Western blotting with rabbit anti-RF1 antibodies and ^125^I-labelled protein A.

Altogether, these results suggested that the *prfA* mutant strains KR4, KR17 and KR19 were deficient in translation termination activity at UAG codons. This hypothesis could be tested using a system described by Curran and Yarus (1989) and developed into a convenient and precise assay system by Poole *et al*. (1995). This system is based on competition between translational termination and ribosomal frameshifting at the frameshift site present in *E. coli prfB*. At this site, an in-frame UGA stop codon in the mRNA is bypassed by a +1 ribosomal frameshift, stimulated by the presence of a Shine–Dalgarno sequence six nucleotides upstream of the stop codon. In the assay system, the RF2 frameshift window is fused to the *malE* gene, expressed from a plasmid. Termination and +1 frameshifting lead to proteins of 43.9 and 52.0 kDa, respectively (Fig. 3A), that are separated by SDS gel electrophoresis and detected by Western blotting, binding of anti-MalE antibody and interaction with ^125^I-labelled Protein A. The ratio of the termination product to the frameshift product is a measure of relative termination efficiency at different stop signals or in different strains.

The activity of the *prfA* mutant strains was tested at UAG stop codons in two contexts: UAGA and UAGU, both less favourable for termination than the most common UAG stop signal, UAGG. Western blots of this assay are shown in Fig. 3B, and the termination rate constants relative to frameshifting in Table 2. Expression of the fusion proteins in the *prfA* mutant strains somewhat inhibited cell growth, which accounts for the reduced amount of fusion protein in these strains; for reasons that are unclear the inhibitory effect was more pronounced with the UAGA-containing fusion. The values found for the termination rate in parental strain MC1061 are close to those reported by Poole *et al*. (1995) but lower than those of Mora *et al*. (2007) in other *E. coli* K12 strains. It should be recalled that strain MC1061 is a streptomycin-dependent strain, grown in the presence of streptomycin, and that this is likely to influence the kinetic parameters for translation and translation termination. As seen in Table 2 (rows 2 and 3), termination rates for all three mutants at both UAGA and UAGU signals were strongly reduced with respect to the wild type by an average factor of about 10.

**Table 2:** Termination rate at UAG codons and quantities of RF1 in *prfA* mutant strains.

	MC1061	KR4	KR17	KR19
RF1 mutation	–	R303H	G121S	G301S
Termination rate at UAGA	1.30	0.18	0.15	0.14
Termination rate at UAGU	1.42	0.16	0.06	0.12
Relative quantity of RF1	100	80	85	85

Data are obtained by quantification of Western blots such as those shown in Fig. 3. Termination rate at UAGA and UAGU stop signals is expressed relative to +1 frameshifting in a frameshift window derived from the RF2 frameshift site. The quantity of RF1 in mutant strains, measured by reaction with anti-RF1 antibodies, is expressed as a percentage of that found in the parental strain MC1061.

Two mechanisms might explain this result: the mutations may affect the folding or stability of RF1, resulting in a strongly reduced amount of RF1 in the cell. Alternatively, the activity of the protein may be reduced compared with that of the normal factor. To resolve this question, the amount of RF1 present in the *prfA* mutant strains was compared with the parental strain by quantitative Western blotting using anti-RF1 antibodies. The results (Fig. 3C, Table 2, row 4) showed that the reduction in the amount of RF1 in the mutant strains was quite modest (15–20%), insufficient to explain the 10-fold drop in termination rate in the cells; the major part in this drop must therefore be due to reduced activity of the mutant factors.

The small decrease in the amount of RF1 found in the mutant strains suggested that the mutant factors were not strikingly less stable than the wild-type factor. This conclusion might however be invalid if an autocontrol mechanism were present in cells, with the consequence that a lowered peptide release activity were to lead to a higher rate of synthesis of the RF. Such an autocontrol mechanism does exist in the case of RF2 in *E. coli* and in about 70% of microorganisms for which genome sequences are known (Baranov *et al*., 2002), based on the competition between termination and frameshifting that we have used to assay termination efficiency. The *prfA* gene is expressed from the promoter of the upstream gene, *hemA*. It has been suggested that *E. coli* and *Salmonella* may possess a mechanism for the autocontrol of RF1 synthesis, dependent upon readthrough of the weak UAGC tetranucleotide stop signal of the *hemA* gene, coupled with a suboptimal ribosome binding site for *prfA* expression (Elliott, 1989). The hypothesis suggests that RF1 synthesis may result to a significant extent from ribosomes that read through the *hemA* stop signal, translate the intergenic region, terminate just short of the *prfA* initiation codon and resume translation without dissociating from mRNA. To test this hypothesis, we constructed translational fusions with *lacZ* extending from the promoter region of *hemA* to codon 13 of *prfA*, analogous to those described by Dahlgren and Ryden-Aulin (2004), and studied the effect on β-galactosidase synthesis of mutating the *hemA* stop signal or introducing mutations in RF1. The results showed that even in the case of the RF1 mutants described in this article, little RF1 is in fact synthesized in *E. coli* by the proposed readthrough mechanism (L. Mora, unpubl. data). These results further confirm that the detected levels of the RF1 mutants reflect a minor effect on stability and therefore that the isolated mutations do not affect substantially the stability of RF1.

### *prfA* mutation leads to hypersensitivity to Kid and RelE toxins

**Fig. 4:**
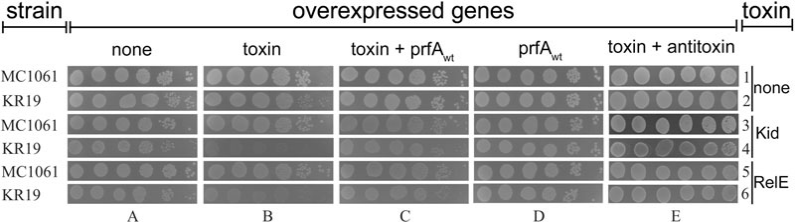
Hypersensitivity of the *prfA* mutant strain KR19 to the Kid and RelE toxins and neutralization by cognate antitoxins and by *prfA*wt overexpression. Parental MC1061 and *prfA* mutant KR19 strains bearing the pNDM220 plasmid vector (lanes 1 and 2) or its derivatives encoding Kid (lanes 3 and 4) and RelE (lanes 5 and 6) toxins were grown exponentially to an *A*_600_ of 0.4 in LB medium. Serial dilutions (10^−1^ steps from left to right) of the different cultures were spotted in LB solidified medium. In (B) and (C), IPTG (100 μM) was included in the plates to induce the expression of the Kid and RelE toxins from the plasmids. In (A) and (D), the same experiment was performed but IPTG was omitted. Specificity controls were carried out by transforming the cells with plasmid pELI08 (*prfA*wt) (C and D) or with the plasmids encoding the respective antitoxins to Kid and RelE, namely plasmids pELI05 (*kis*) and pELI09 (*relB*) (E). The plates were incubated at 30°C and photographs were taken after 18 h incubation.

Since RF1 was shown to protect from RNA cleavage by the RelE toxin *in vitro* (Pedersen *et al*., 2003), we hypothesized that the mutant RF1 strains should be particularly responsive to the activity of RelE *in vivo*. For this analysis we selected the *prfA* mutant KR19 as this mutant showed the lowest overall termination efficiency in the assays (see Table 2). We produced the RelE protein in this mutated *prfA* strain and compared the levels of cytotoxicity with those achieved in a wild-type *E. coli* background. To gain insight into the mechanism of RF1-dependent protection, we performed a parallel control experiment in which the Kid toxin was produced in place of RelE. Figure 4 shows the cytotoxic effects of both Kid and RelE proteins over the wild type, MC1061, and the mutant strain KR19 bearing the plasmids pNDM220 (vector), pELI06 (*kid*) or pELI07 (*relE*). Figure 4A and D shows that the growth of the strains in the absence of induction of the toxins is unaffected. The partial induction of toxins Kid or RelE with a less-than-saturating concentration of IPTG (100 μM) inhibited cell growth in *prfA* mutant strains but not in the wild-type strain MC1061 (Fig. 4B, rows 4 and 6 compared with rows 3 and 5). This suggested that KR19 was hypersensitive to both toxins. When this strain was transformed with pELI08 (*prfA*wt), normal sensitivity to toxins was restored (Fig. 4C, rows 3–6), suggesting a specific effect of the mutations in *prfA* on the hypersensitivity to the toxins. The growth inhibition of the *prfA* mutants by the Kid and RelE toxins was neutralized when their respective antitoxin genes *kis* and *relB* were coexpressed (Fig. 4E, rows 3–6). Altogether these experiments brought to light a novel and specific functional interplay between RF1 and the Kid and RelE toxins.

**Fig. 5:**
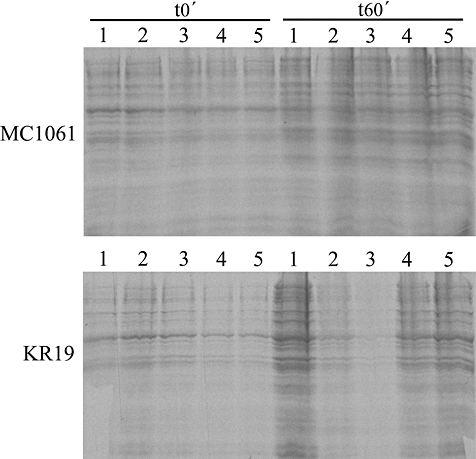
Inhibition of protein synthesis *in vivo* by the Kid and RelE toxins. Strains MC1061 (top) or KR19 (bottom) were transformed with plasmids as indicated: lane (1) plasmid vector pNDM220, lane (2) pSS100 (*kid*), lane (3) pELI07 (*relE*), lane (4) pSS100 (*kid*) and pELI05 (*kis*), lane (5) pELI07 (*relE*) and pELI09 (*relB*). When relevant, expression of the toxin and antitoxin genes was triggered by adding IPTG (100 μM) and arabinose (0.5%) to the medium respectively. The proteins synthesized at time 0 or 60 min after the induction of the toxin and antitoxin genes were fractionated by SDS-PAGE (12.5%) and identified by autoradiography as described in *Experimental procedures*.

Both RelE and Kid toxins are inhibitors of protein synthesis. We therefore evaluated the effect of these toxins on the *de novo* protein synthesis in the *prfA* mutant and wild-type strains at the suboptimal induction conditions mentioned above. The results (Fig. 5) indicated that indeed the RelE and Kid toxins inhibited protein synthesis in the *prfA* mutant but not in the wild-type strain. The enhanced inhibition of protein synthesis by Kid and RelE was prevented when their cognate Kis and RelB antitoxins were expressed in the cells, respectively (Fig. 5), indicating as in the previous assay that the inhibitory effect on protein synthesis is specific to the activity of the toxins. Protection by the RF1wt protein in this assay was less efficient, which is likely a reflection of the more specific interaction of a toxin with its cognate antitoxin (data not shown). Altogether, the increased efficiency in protein synthesis inhibition was consistent with the enhanced cytotoxic effect displayed by the Kid and RelE proteins in the mutant *prfA* background.

**Fig. 6:**
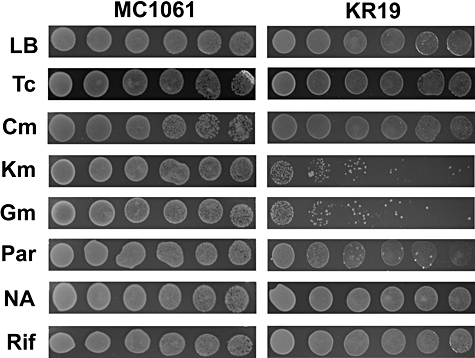
Sensitivity assays of the KR19 (prfA301) and the parental strain MC1061 to different antibiotics. Parental MC1061 and *prfA* mutant KR19 strains were grown at 30°C to an *A*_600_ of 0.4 in LB medium. Serial dilutions (10^−1^ steps from left to right) of the different cultures were spotted in LB solidified medium (lane LB) as a positive control or in the same medium supplemented with the different antibiotics: tetracycline (Tc, 0.1 μg ml^−1^), chloramphenicol (Cm, 0.5 μg ml^−1^), kanamycin (Km, 1.0 μg ml^−1^), gentamicin (Gm, 0.5 μg ml^−1^), paromomycin (Par, 1.0 μg ml^−1^), nalidixic acid (NA, 1.0 μg ml^−1^) and rifampycin (Rif, 1.0 μg ml^−1^). Concentrations of the different antibiotics close to their minimal inhibitory concentrations were used. The plates were incubated at 30°C and photographs were taken after 18 h incubation.

To test whether the extra-sensitivity to the toxins of the *prfA* mutants was an indirect consequence of a general stress on the translation apparatus we first compared the sensitivity of the wild type and mutant strain to antibiotics acting on translation, such as chloramphenicol or tetracycline, inhibitors of the peptidyl transferase activity or of aminoacyl tRNA binding respectively (Chopra and Roberts, 2001; Hansen *et al*., 2003). The sensitivity to different aminoglycosides (kanamycin, gentamicin and paromomycin) was also tested. These family of antibiotics interact at the A site on the 30S subunit of the ribosome affecting codon–anticodon recognition and translocation of tRNA between the A and P sites (Kotra *et al*., 2000). We obtained the following results (see Fig. 6): (i) there were no differences in the sensitivity of the wild-type and *prfA* strains to chloramphenicol and tetracycline, thus ruling out increased sensitivity to Kid and RelE as a circunstancial consequence of a general stress on the translation apparatus imposed by the *prfA* mutations, and (ii) the mutant strain was more sensitive than the parental one to kanamycin, and to the other aminoglycosides tested. This is somehow an expected result as these antibiotics interact with a region that is overlapping with the domain of interaction of RF1 (see *Discussion*).

As an additional control to test possible unspecific effects of the *prfA* mutation on other cellular processes, we used Nalidixic acid (an inhibitor of DNA Gyrase and an inductor of the SOS response) or Rifampycin (an inhibitor of RNA polymerase and therefore of transcription). No differences between wild type and *prfA* mutants to the sensitivity to these inhibitors were found (Fig. 6). This further supports the specificity of the increased sensitivity to the RelE and Kid toxin observed in the mutants.

## Discussion

**Fig. 7:**
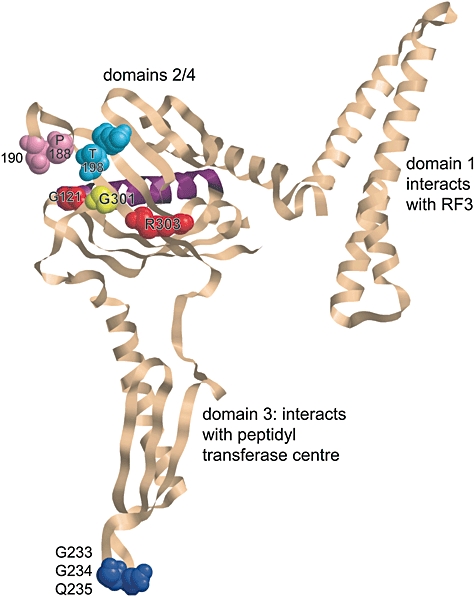
Positions of the mutations in RF1 in relation to domains and sequence motifs of functional importance. The positions of residues G121, G301 and G303 for which mutants are found are shown space-filled in red (G121 and R203) or yellow (G301) on a model of the open form of *Thermotoga maritima* RF1 in ribbon representation (Vestergaard *et al*., 2005). The α5 helix, which has G121 at the tip, is shown in violet. The PxT motif implicated in recognition of the stop codon (Ito *et al*., 2000) is shown space-filled in pink, and residues T198 and Q185 interacting with the third stop codon nucleotide (Laurberg *et al*., 2008) are space-filled in light blue. The GGQ motif that interacts with the peptidyl transferase centre and triggers peptidyl-tRNA hydrolysis is shown space-filled in blue.

**Fig. 8:**
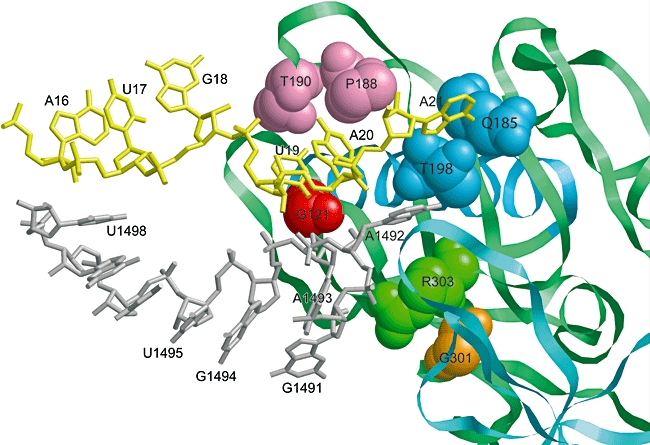
Ribosomal and mRNA components near to mutant residues in RF1. Components near residues G121, G301 and R303 in *E. coli* RF1 are shown, based on the 3.2 Å resolution crystal structure of Laurberg *et al*. (2008) of *Thermus thermophilus* RF1 bound to homologous 70S ribosomes in the presence of a short mRNA (5′-GGC AAG GAG GUA AAA A_16_U_17_G_18_**U_19_A_20_A_21_** AAA AAA-3′; stop codon in bold; nucleotides 16–21 are shown in yellow) and P- and E-site bound tRNA^Met^_f_. The RF1 polypeptide is shown as a green ribbon (except for helix α5, in blue), with mutant residues space-filled (G121: red, G301: orange, R303: green); residues are numbered as in *E. coli* RF1. Residues in RF1 involved in stop codon recognition are also shown space-filled (P188 and T190: rose, Q185 and T198: blue). Nucleotides 1491–1498 in 16S rRNA are shown in grey, and ribosomal protein S12 is shown as a turquoise ribbon. See Table 3 for more information about near neighbours of the mutant residues in RF1.

In this article we report the isolation and characterization of novel mutations in *prfA* affecting the translation termination factor RF1. As previously described these mutations were selected by means of a novel TA system-based genetic screen leading to the efficient counter-selection of cells with intact translation termination capacities. The three mutant residues isolated in RF1 are grouped quite closely together and are nearby the region of RF1 known to be involved in stop codon recognition (Fig. 7). This includes a tripeptide motif (PxT, 188–190 in RF1, SPF, 205–207 in RF2) (Ito *et al*., 2000) important in the recognition of the first and second stop codon nucleotides (Laurberg *et al*., 2008) and amino acid residues at the tip of helix α5 (shown in blue in Fig. 7) in RF1 and RF2, which help discriminate against purines at the constant first position U of stop codons (Petry *et al*., 2005; Laurberg *et al*., 2008). One of the mutations, G121, is present at the tip of this helix, and is within about 6 Å of the first two nucleotides, U4 and A5 of the mRNA stop codon (Fig. 8, Table 3). Mutation R303 is close to A1492 and A1493 in 16S rRNA, involved both in recognition of sense codons in the A site by tRNA (Ogle *et al*., 2001; Ogle and Ramakrishnan, 2005) and in translation termination (Laurberg *et al*., 2008), as well as to other adjacent nucleotides. Both R303 and G301 are close to ribosomal protein S12 (Fig. 8, Table 3). Mutations in S12 are known to affect the accuracy of stop codon recognition. Thus all three mutations are situated close to the stop codon recognition site, which is consistent with their effect on the efficiency of translation termination mediated by RF1. It would be of interest to study their effect on the accuracy of stop codon recognition.

**Table 3:** Components of mRNA, 16S rRNA and proteins of the 30S ribosomal subunit within about 6 Å of the mutant residues in RF1.

Mutant residue	mRNA	16S rRNA	30S proteins
G121	U4, A5	A1493, G1494	
G301			S12: T43
R303		A1491, A1492	S12: T43, P44

The nearby components were identified using the structure file of the RF1:70S ribosomal complex (Laurberg *et al*., 2008). See also Fig. 8.

These novel *prfA* mutants show increased sensitivity to the RelE and Kid toxins. This phenomenon, detected both in growth and in protein synthesis assays, and the suppression of this sensitivity by the overproduction of prfAwt *in vivo* revealed an unexpected confluence in the pathway of action of these two toxins. Previous reports showed that the RelE toxin acts as an endoribonuclease in concerted action with the ribosome to cleave at the UAG termination codon at the A site of the ribosome, thus leading to translation inhibition. Consistent with this it was found that RF1 prevents RNA cleavage by this toxin (Pedersen *et al*., 2003). RelE seems to have a shape similar to the C-terminal region of translation elongation factor EF-G (Takagi *et al*., 2005), which could explain its access to the UAG codon in the A site of the ribosome. The protection conferred by RF1 against the action of RelE likely reflects a competition for the same substrate. The mutations could affect this competition ultimately favouring the action of the toxin. A similar explanation could account for the extra-sensitivity of the *prfA* mutant to paromomycin and other aminoglycosides, which interact with the 30S subunit of the ribosome at a region overlapping with the site of interaction of RF1. Paromomycin binding to 30S ribosomes directly displaces A1492 and A1493 from 16S rRNA helix 44 to a bulged-out state characteristic of ribosomes containing cognate tRNA in the A site (Ogle *et al*., 2001). The modes of binding of kanamycin and gentamicin to the ribosome are similar (Francois *et al*., 2005). The extrahelical position of A1493 sterically impedes RF1 binding (Laurberg *et al*., 2008), explaining the competitive inhibition of termination by paromomycin (Youngman *et al*., 2007). These aminoglycosides therefore introduce perturbations into the same region of the decoding site as the RF1 mutations we describe here, suggesting that the increased sensitivity of the mutant strains to the antibiotics is due to an accumulation of factors interfering with stop codon recognition by RFs.

Unlike RelE, the Kid and ChpAK (MazF) toxins are able to cleave RNA in the absence of ribosomes (Zhang *et al*., 2003; 2004; Munoz-Gomez *et al*., 2004; 2005) following a mechanism similar to the one described for RNase A and RNase T1 (Christensen *et al*., 2003; Pedersen *et al*., 2003; Kamphuis *et al*., 2006). Since the structure of the Kid protein is clearly different from that of RelE, similarities in their mechanisms to access the A site of the ribosome seem unlikely. It is noteworthy however that the Kid toxin can cleave the minimal substrate UpA which is part of the termination codon UAG/A (Kamphuis *et al*., 2006). It remains to be seen if the hypersensitivity to Kid observed in the *prfA* mutants could be due to interactions between Kid and RF1, which remain to be elucidated. Alternatively, in view of the fact that reduced activity of the RF1 can induce ribosome stalling at the stop codon and subsequent mRNA cleavage (Li *et al*., 2007), the increased toxicity of the *prfA* mutants to the Kid toxin could also be due to an enhancement of this stalling. Whatever the mechanism, it seems clear that the increased sensitivity of the *prfA* mutants to aminoglycosides and to the Kid and RelE toxins is an specific effect of these mutations rather than the unspecific consequence of a general stress on translation induced by them. This result provided the first evidence for the involvement of RF1 in the pathway of Kid toxicity.

The new *prfA* mutants contributed to showing that a post-transcriptional mechanism is not involved in maintaining the levels of the RF1 in the cells. The mutants could be used to further explore the contribution of new residues close to the RF decoding region in the recognition of the termination codons and/or in the transmission of the signal that activates the transpeptidase of RF1 at the PTC distal site. In addition, the experiments reported provide the first *in vivo* evidence for a functional interaction between the RelE toxin and the RF1 protein. Furthermore, evidence is provided for a role of translation termination factors in the cytotoxicity pathway of the Kid toxin. The results in turn reveal an unexpected functional confluence between two distinct families of bacterial toxins, namely the RelE and Kid families. Similarities and differences in these pathways remain to be fully established. The hypersensitivity of the *prfA* mutants to the RelE and Kid toxins also opens the possibility of exploring their effects on other toxins belonging to the *kis–kid/ccd* and the *relBE/parDE* superfamilies (Gerdes *et al*., 2005). These include toxins targeting DNA gyrase such as CcdB and ParE in addition to toxins which acts on RNA as Kid and RelE. In theory it can be anticipated that the *prfA* mutants should also be extra-sensitive to the RNase-type toxins of the family but not to the toxins targeting the DNA Gyrase. Finally, the results and methods reported here provide a rationale for the design of screening approaches to isolate inhibitors of RF1 activity. Such compounds would likely potentiate the antibacterial activity of TA systems. Most interestingly, some antibiotics have been reported to specifically activate TA systems (Sat *et al*., 2001). In this respect as well, compound drugs inhibiting RF1 activity would be particularly appealing as they would provide a back-up induction of TA toxicity in antibiotic-resistant strains. Novel therapeutic alternatives against drug-resistant pathogens are urgently needed and the work reported here certainly opens new avenues towards that goal.

## Experimental procedures

### Bacteria strains

*Escherichia coli* K-12 strains used in this work are described in Table 4.

**Table 4:** Strains used in this work.

Strain	Genotype	Reference
BL21 (DE3)	F^-^*ompT hsdS*_*B*_ (rB^-^ mB^-^) *gal dcm*λDE3	Novagen
MC1061	F^-^*araD139 galE15 galK16 rpsL hsdR2* (rK− mK+) *mcrA mcrB1*	*E. coli* Genetic Stock Center
KR19 (KR2)	MC1061 *prfA301*	This work
KR4	MC1061 *prfA303*	This work
KR17	MC1061 *prfA121*	This work
MRA8	*prfA1(ts)*	Zhang *et al*. (1994)
W3110	F^-^λ^-^*rph-1* INV(*rrnD*, *rrnE*)	Bachmann (1972)

### Genetic screen

*Escherichia coli* K-12 bacteria (strain MC1061) were subjected to mutagenesis following treatment with 2-aminopurine as described by Miller (1972). The treated bacteria were made electrocompetent and were electroporated with plasmid pKK1120 using a Bio-Rad MicroPulser^TM^ and following the manufacturer's instructions. The cells were allowed to recover by shaking in SOC (Hanahan, 1983) medium for 1 h at 37°C. After a brief centrifugation, the cells were re-suspended in LB medium (Miller, 1972) containing tetracycline (10 μg ml^−1^) and incubated at 37°C with aeration for 14 h. After two washes in saline, the cells were rendered electrocompetent as described above and were electroporated with plasmid pAB1120. The cells were recovered in SOC medium and were spread on LB agar plates containing kanamycin (50 μg ml^−1^) and streptomycin (200 μg ml^−1^). The plates were incubated at 37°C for more than 18 h.

### Plasmid construction

Plasmids used and constructed in this work are listed in Table 1. To construct the pBAD18-derivative plasmids (Table 1), PCR fragments were generated using primers PRL (+) (5′-GACAGCTAGCGGCTGGAGTA-3′; NheI site underlined) and PRR (−) (5′-GGTAGCATGCTCCAGCAGGATTTC-3′; SphI site underlined) and genomic DNA originated from the wild-type strain as well as from the different mutants as templates. The resulting fragments were introduced into pBAD18 vector digested with NheI and SphI, to yield pELI01, pELI02, pELI03 and pELI04 plasmids.

Plasmids pELI05 and pELI06 (Table 1) were constructed by generating PCR fragments encoding the *kis* and *kis74* alleles using primers KIS5N (+) (5′-GGCGAGCATATGGAGGTGAAGAAT-3′, NdeI site underlined) and KIS CTR (−) (5′-GCTGGATCCTCAGATTTCCTCCTGAC-3′ which introduces a BglII site) and plasmids pET80 and pAB1120, respectively, as DNA templates. PCR products bearing the *kis* alleles were introduced into pFUS2 after being digested with NdeI and BglII, resulting in the pELI05 and pELI06 plasmids.

To construct the pELI07 plasmid, a PCR fragment encoding *relE* was generated using primers relE1B and relE2 described in Gotfredsen *et al*. (1998) and genomic DNA from W3110 as template. The PCR product containing *relE* was introduced into pNDM220 after being digested with BamHI and SalI, yielding the pELI07 plasmid.

The pELI08 plasmid was constructed using a fragment containing the wild-type *prfA* gene generated from the digestion of pELI02 with HindIII and NsiI inserted into the pFUS2 vector cleaved with the same enzymes.

The pELI09 plasmid was constructed by introducing a PCR fragment encoding *relB* into pFUS2 after a double digestion with NdeI and HindIII. The PCR fragment was generated using W3110 as DNA template and degenerated primers relB1 and relB2 described in Gotfredsen and Gerdes (1998), relB1 (5′-CCCCCGCATATGTAATTACAAGAGGTGTAAGAC-3′) and relB2 (5′-CCCCCTCGAGAAGCTTCAGAGTTCATCCAGCGTCACACGTACT-GG-3′).

pKK1120 (*p15a*, *kis74*, *kid*) was constructed by ligating the 1.2 kb FspI–EcoRI fragment of pAB1120 containing the *kis74 (amber)-kid* mutant operon and the 3.8 kb EcoRI–ScaI vector fragment from pACYC184.

### Western blotting for readthrough assay

Cultures growing overnight in LB media were diluted 1:100 and induced with 1% arabinose when the OD_600_ reached 0.1. At OD_600_ 0.3, aliquots of 2 ml were centrifuged and the pellet was re-suspended in 60 μl of Laemmli buffer (Laemmli, 1970) and boiled for 10 min in order to obtain the cell lysates. Sodium dodecyl sulphate polyacrylamide gel electrophoresis (SDS-PAGE) was performed with a Hoefer SE 260 apparatus in the presence of running buffer [Tris 0.025 M, glycine 0.192 M, SDS 0.1% (w/v) pH 8.5]. The separating gel contained 15% (w/v) of acrylamide. Following electrophoresis, proteins were transferred onto polyvinylidene difluoride (PVDF) membrane (Sequi-Blot^TM^ Bio-Rad Laboratories) using a Trans-Blot SD Semi-Dry Transfer Cell apparatus (Bio-Rad Laboratories) at a constant current of 12 V for 90 min at room temperature. PVDF membrane was blocked overnight in 137 mM NaCl, 0.1% (w/v) Tween 20 and 20 mM Tris/HCl, pH 7.5 (buffer TBST), at room temperature. The membrane was incubated for 1 h with the anti-Kis antibody diluted ×2000 in buffer TBST. The membrane was then washed four times by gentle agitation in buffer TBST for 30 min, incubated for 1 h with an anti-rabbit Ig linked to horseradish peroxidase and diluted ×10 000 (Amersham Biosciences) in buffer TBST. Subsequently the membrane was washed four times in buffer TBST for 30 min, the labelled Kis were revealed with enhanced chemiluminescence (ECL) detection system (Amersham Biosciences). Agfa films were exposed to the membranes and developed.

### Assay for termination efficiency *in vivo*

Plasmids expressing MalE, fused to LacZα through a window containing the RF2 frameshift site, were as described by Poole *et al*. (1995). Parental and *prfA* mutant strains were transformed and grown in LB medium containing 200 μg ml^−1^ streptomycin and 200 μg ml^−1^ ampicillin to an OD_600_ of 0.5 and induced with 1 mM IPTG for 2 h. Cell proteins were solubilized in SDS gel loading buffer and 3 or 6 μl aliquots were analysed by SDS-PAGE electrophoresis as described by Poole *et al*. (1995). Transfer to nitrocellulose membranes (Hybond C Super, GE Healthcare) and Western blotting with anti-MalE antibodies (Anti-MBP-antiserum, NEB) diluted ×5000 were performed as described by Sambrook *et al*. (1989), using ^125^I-labelled protein A (GE Healthcare). Radioactivity was measured using a phosphorimager (Molecular Dynamics).

### Western blotting of MalE fusion proteins

Cultures of strains transformed with plasmids expressing the MalE fusion proteins were grown in LB medium, 200 μg ml^−1^ streptomycin, 200 μg ml^−1^ ampicillin and induced with 1 mM IPTG as described for *in vivo* termination efficiency experiments. Aliquots of 1 ml of culture were centrifuged and the cells were lysed for 10 min at 100°C in 200 μl of lysis buffer (50 mM Tris-HCl, pH 6.8, 100 mM DTT, 2% SDS, 0.1% bromophenol blue, 10% glycerol). Aliquots of 20 μl in the case of the parental strain MC1061 and volumes containing equivalent amounts of total protein in the case of the mutant strains were applied to 10% polyacrylamide gels for separation as described by Laemmli (1970). Transfer to nitro-cellulose membranes, Western blotting with antibodies and quantification were performed as described above. Western blots were performed with rabbit anti-RF1 diluted ×5000 commercially prepared from pure factor.

### Complementation assays

Cultures of strain MRA8 (*prfA1ts*) bearing pBAD-derivative plasmids (*prfA* wild type and mutants) were grown overnight at 30°C prior to being diluted 1:100 into two different aliquots of fresh medium (LB medium; ampicillin, 25 μg ml^−1^), with or without arabinose (0.2% v/v). The cultures were transferred to 42°C, and the OD_600_ of both cultures was monitored every 20 min over a period of 160 min.

### Toxicity assays

MC1061 wild-type and *prfA* mutant strains bearing pNDM220-derivative plasmids containing or not pELI08 were grown at 30°C to mid-exponential phase in LB broth plus ampicillin (50 μg μl^−1^) and kanamycin (as needed, 50 μg μl^−1^). The same strains containing the pFUS2 derivatives containing the antitoxin genes were grown in a similar way. Aliquots of the different cultures and serial dilutions (10^−1^ steps) were spotted onto plates of the same solidified medium containing or not IPTG 100 μM as inducer of the toxins and were incubated at 30°C for 18 h. Antitoxins were induced using 0.5% arabinose. A semi-quantitative assessment of the relative levels of toxicity was derived from the differential dilution factor obtained for two spots presenting an equivalent number of colonies. Similar procedures were used to test the differential sensitivity of KR19 and MC1061 strains to the different antibiotics.

### *In vivo de novo* synthesis of ^35^S-labelled proteins

One millilitre of cultures of wild-type and mutant strains KR19 bearing pSS100 (*kid*) plus or not pELI08 (*prfAwt*) or pELI05 (*kis*), or KR19 bearing pELI07 (*relE*) plus or not pELI08 (*prfAwt*) or pELI09 (*relB*) were labelled with 14.3 μCi of [^35^S]Met/Cys (80:20, Amersham Biosciences) for 2 min at 37°C, then 1 mg of unlabelled methionine was added and incubation was continued for 8 min at 37°C. Cells were centrifuged at 20 000 *g* for 2 min at 4°C, the pellet was washed with a buffer containing KCl 3 mM, NaCl 68 mM, KH_2_PO_4_ 1.5 mM and NaH_2_PO_4_ 9 mM and re-suspended in 10 μl of protein loading buffer 2×[0.05 M Tris-HCl pH 6.8, 10% (w/v) SDS, 0.01 M EDTA 25% (w/v), Bromophenol blue 0.5 g l^−1^ and β-mercaptoethanol 5% (v/v)]. Proteins were separated by 12.5% SDS-PAGE and the radiolabelled products were identified by autoradiography.

## References

[b1] Bachmann BJ (1972). Pedigrees of some mutant strains of *Escherichia coli* K-12. Bacteriol Rev.

[b2] Baranov PV, Gesteland RF, Atkins JF (2002). Release factor 2 frameshifting sites in different bacteria. EMBO Rep.

[b3] Bernard P, Couturier M (1992). Cell killing by the F plasmid CcdB protein involves poisoning of DNA–topoisomerase II complexes. J Mol Biol.

[b4] Bravo A, Ortega S, de Torrontegui G, Diaz R (1988). Killing of *Escherichia coli* cells modulated by components of the stability system ParD of plasmid R1. Mol Gen Genet.

[b5] Chopra I, Roberts M (2001). Tetracycline antibiotics: mode of action, applications, molecular biology, and epidemiology of bacterial resistance. Microbiol Mol Biol Rev.

[b6] Christensen SK, Pedersen K, Hansen FG, Gerdes K (2003). Toxin–antitoxin loci as stress-response-elements: ChpAK/MazF and ChpBK cleave translated RNAs and are counteracted by tmRNA. J Mol Biol.

[b7] Condon C (2006). Shutdown decay of mRNA. Mol Microbiol.

[b8] Curran JF, Yarus M (1989). Rates of aminoacyl-tRNA selection at 29 sense codons *in vivo*. J Mol Biol.

[b9] Dahlgren A, Ryden-Aulin M (2004). Effects of two cis-acting mutations on the regulation and expression of release factor one in *Escherichia coli*. Biochimie.

[b10] Elliott T (1989). Cloning, genetic characterization, and nucleotide sequence of the *hemA-prfA* operon of *Salmonella typhimurium*. J Bacteriol.

[b11] Francois B, Russell RJ, Murray JB, Aboul-ela F, Masquida B, Vicens Q, Westhof E (2005). Crystal structures of complexes between aminoglycosides and decoding A site oligonucleotides: role of the number of rings and positive charges in the specific binding leading to miscoding. Nucleic Acids Res.

[b12] Freistroffer DV, Pavlov MY, MacDougall J, Buckingham RH, Ehrenberg M (1997). Release factor RF3 in *E. coli* accelerates the dissociation of release factors RF1 and RF2 from the ribosome in a GTP-dependent manner. EMBO J.

[b13] Freistroffer DV, Kwiatkowski M, Buckingham RH, Ehrenberg M (2000). The accuracy of codon recognition by polypeptide release factors. Proc Natl Acad Sci USA.

[b14] Frolova LY, Tsivkovskii RY, Sivolobova GF, Oparina NY, Serpinsky OI, Blinov VM (1999). Mutations in the highly conserved GGQ motif of class 1 polypeptide release factors abolish ability of human eRF1 to trigger peptidyl-tRNA hydrolysis. RNA.

[b15] Gao H, Zhou Z, Rawat U, Huang C, Bouakaz L, Wang C (2007). RF3 induces ribosomal conformational changes responsible for dissociation of class I release factors. Cell.

[b16] Gerdes K, Christensen SK, Lobner-Olensen A (2005). Prokaryotic toxin–antitoxin stress response loci. Nat Rev Microbiol.

[b17] Gotfredsen M, Gerdes K (1998). The *Escherichia coli relBE* genes belong to a new toxin–antitoxin gene family. Mol Microbiol.

[b18] Guzman LM, Belin D, Carson MJ, Beckwith J (1995). Tight regulation, modulation, and high-level expression by vectors containing the arabinose PBAD promoter. J Bacteriol.

[b19] Hanahan D (1983). Studies on transformation of *Escherichia coli* with plasmids. J Mol Biol.

[b20] Hansen JL, Moore PB, Steitz TA (2003). Structures of five antibiotics bound at the peptidyl transferase center of the large ribosomal subunit. J Mol Biol.

[b21] Hayes CS, Sauer RT (2003). Cleavage of the A site mRNA codon during ribosome pausing provides a mechanism for translational quality control. Mol Cell.

[b22] Ito K, Uno M, Nakamura Y (2000). A tripeptide ‘anticodon’ deciphers stop codons in messenger RNA. Nature.

[b23] Kamphuis MB, Bonvin AM, Monti MC, Lemonnier M, Munoz-Gomez A, van den Heuvel RH (2006). Model for RNA binding and the catalytic site of the RNase Kid of the bacterial parD toxin–antitoxin system. J Mol Biol.

[b24] Karimi R, Pavlov MY, Buckingham RH, Ehrenberg M (1999). Novel roles for classical factors at the interface between translation termination and initiation. Mol Cell.

[b25] Kotra LP, Haddad J, Mobashery S (2000). Aminoglycosides: perspectives on mechanisms of action and resistance and strategies to counter resistance. Antimicrobial Agents Chemother.

[b26] Laemmli UK (1970). Cleavage of structural proteins during the assembly of the head of bacteriophage T4. Nature.

[b27] Laurberg M, Asahara H, Korostelev A, Zhu J, Trakhanov S, Noller HF (2008). Structural basis for translation termination on the 70S ribosome. Nature.

[b28] Lemonnier M, Bouet JY, Libante V, Lane D (2000). Disruption of the F plasmid partition complex *in vivo* by partition protein SopA. Mol Microbiol.

[b29] Li X, Yokota T, Ito K, Nakamura Y, Aiba H (2007). Reduced action of polypeptide release factors induces mRNA cleavage and tmRNA tagging at stop codons in *Escherichia coli*. Mol Microbiol.

[b30] Miller J (1972). Experiments in Molecular Genetics.

[b31] Moore SD, Sauer RT (2007). The tmRNA system for translational surveillance and ribosome rescue. Annu Rev Biochem.

[b32] Mora L, Heurgue-Hamard V, de Zamaroczy M, Kervestin S, Buckingham RH (2007). Methylation of bacterial release factors RF1 and RF2 is required for normal translation termination *in vivo*. J Biol Chem.

[b33] Munoz-Gomez A (2004). Identificación y caracterización de la actividad RNasa de las toxinas bacterianas Kid y ChpAK.

[b34] Munoz-Gomez AJ, Santos-Sierra S, Berzal-Herranz A, Lemonnier M, Diaz-Orejas R (2004). Insights into the specificity of RNA cleavage by the *Escherichia coli* MazF toxin. FEBS Lett.

[b35] Munoz-Gomez AJ, Lemonnier M, Santos-Sierra S, Berzal-Herranz A, Diaz-Orejas R (2005). RNase/anti-RNase activities of the bacterial parD toxin–antitoxin system. J Bacteriol.

[b36] Ogle JM, Ramakrishnan V (2005). Structural insights into translational fidelity. Annu Rev Biochem.

[b37] Ogle JM, Brodersen DE, Clemons WM, Tarry MJ, Carter AP, Ramakrishnan V (2001). Recognition of cognate transfer RNA by the 30S ribosomal subunit. Science.

[b38] Pedersen K, Zavialov AV, Pavlov MY, Elf J, Gerdes K, Ehrenberg M (2003). The bacterial toxin RelE displays codon-specific cleavage of mRNAs in the ribosomal A site. Cell.

[b39] Petry S, Brodersen DE, Murphy FVT, Dunham CM, Selmer M, Tarry MJ (2005). Crystal structures of the ribosome in complex with release factors RF1 and RF2 bound to a cognate stop codon. Cell.

[b40] Poole ES, Brown CM, Tate WP (1995). The identity of the base following the stop codon determines the efficiency of *in vivo* translational termination in *Escherichia coli*. EMBO J.

[b41] Ronen A (1980). 2-Aminopurine. Mutat Res.

[b42] Ryden SM, Isaksson LA (1984). A temperature-sensitive mutant of *Escherichia coli* that shows enhanced misreading of UAG/A and increased efficiency for some tRNA nonsense suppressors. Mol Gen Genet.

[b43] Sambrook J, Fristsch EF, Maniatis T (1989). Molecular Cloning.

[b44] Santos-Sierra S, Pardo-Abarrio C, Giraldo R, Diaz-Orejas R (2002). Genetic identification of two functional regions in the antitoxin of the parD killer system of plasmid R1. FEMS Microbiol Lett.

[b45] Sat B, Hazan R, Fisher T, Khaner H, Glaser G, Engelberg-Kulka H (2001). Programmed cell death in *Escherichia coli*: some antibiotics can trigger mazEF lethality. J Bacteriol.

[b46] Scolnick E, Tompkins R, Caskey T, Nirenberg M (1968). Release factors differing in specificity for terminator codons. Proc Natl Acad Sci USA.

[b47] Sunohara T, Jojima K, Tagami H, Inada T, Aiba H (2004). Ribosome stalling during translation elongation induces cleavage of mRNA being translated in *Escherichia coli*. J Biol Chem.

[b48] Takagi H, Kakuta Y, Okada T, Yao M, Tanaka I, Kimura M (2005). Crystal structure of archaeal toxin–antitoxin RelE-RelB complex with implications for toxin activity and antitoxin effects. Nat Struct Mol Biol.

[b49] Trobro S, Aqvist J (2007). A model for how ribosomal release factors induce peptidyl-tRNA cleavage in termination of protein synthesis. Mol Cell.

[b50] Vestergaard B, Sanyal S, Roessle M, Mora L, Buckingham RH, Kastrup JS (2005). The SAXS solution structure of RF1 differs from its crystal structure and is similar to its ribosome bound cryo-EM structure. Mol Cell.

[b51] Youngman EM, He SL, Nikstad LJ, Green R (2007). Stop codon recognition by release factors induces structural rearrangement of the ribosomal decoding center that is productive for peptide release. Mol Cell.

[b52] Zavialov AV, Buckingham RH, Ehrenberg M (2001). A posttermination ribosomal complex is the guanine nucleotide exchange factor for peptide release factor RF3. Cell.

[b53] Zavialov AV, Mora L, Buckingham RH, Ehrenberg M (2002). Release of peptide promoted by the GGQ motif of class 1 release factors regulates the GTPase activity of RF3. Mol Cell.

[b54] Zhang J, Zhang Y, Zhu L, Suzuki M, Inouye M (2004). Interference of mRNA function by sequence-specific endoribonuclease PemK. J Biol Chem.

[b55] Zhang S, Ryden-Aulin M, Kirsebom LA, Isaksson LA (1994). Genetic implication for an interaction between release factor one and ribosomal protein L7/L12 *in vivo*. J Mol Biol.

[b56] Zhang Y, Zhang J, Hoeflich KP, Ikura M, Qing G, Inouye M (2003). MazF cleaves cellular mRNAs specifically at ACA to block protein synthesis in *Escherichia coli*. Mol Cell.

